# Long‐term effects of prophylactic MgSO_4_ in maternal immune activation rodent model at adolescence and adulthood

**DOI:** 10.1002/jnr.25148

**Published:** 2022-11-26

**Authors:** Fadwa Dabbah‐Assadi, Sally Rashid, Idit Golani, Alon Rubinstein, Ravid Doron, David Alon, Eilam Palzur, Ron Beloosesky, Alon Shamir

**Affiliations:** ^1^ Psychobiology Research Laboratory Mazor Mental Health Center Akko Israel; ^2^ The Ruth and Bruce Rappaport Faculty of Medicine Technion—Israel Institute of Technology Haifa Israel; ^3^ Department of Biotechnology Engineering ORT Braude College Karmiel Israel; ^4^ Faculty of Medicine in the Galilee Bar‐Ilan University Zefat Israel; ^5^ Department of Education and Psychology the Open University Raanana Israel; ^6^ The Neuroscience Laboratory Galilee Medical Center Research Institute Nahariya Israel; ^7^ Department of Obstetrics and Gynecology Rambam Medical Center Haifa Israel

**Keywords:** animal behavior, LPS, magnesium sulfate, maternal immune activation (MIA), neuroprotection, NRG–Erbb4 signaling

## Abstract

The effects of MgSO_4_ as an anti‐inflammatory agent in pregnant women have been investigated in the last few years. Infections can cause an inflammatory reaction involving the placenta membranes and amniotic cavity. They may have short‐term effects on the mother and her fetuses, like preterm birth, cerebral palsy, and developmental delay. Despite the alleged advantages of MgSO_4_ as a neuroprotective agent in the preterm brain, the long‐term molecular and behavioral function of MgSO_4_ has not been fully elucidated. Here, we investigated the long‐term effect of antenatal MgSO_4_, during late gestation, on offspring's behavior focusing on cognitive function, motor activity, and social cognition in adolescence and adulthood, and explored its influence on brain gene expression (e.g., ErbB signaling, pro‐inflammatory, and dopaminergic markers) in adulthood. A significant abnormal exploratory behavior of offspring of MgSO_4_‐treated dams was found compared to the control group in both adolescence and adulthood. Furthermore, we found that adult females exposed to MgSO_4_ under inflammation displayed working and recognition memory impairment. A reduction in IL‐6 expression was detected in the prefrontal cortex, and hippocampus specimens derived from LPS–Mg‐treated group. In contrast, an imbalanced expression of dopamine 1 and 2 receptors was detected only in prefrontal cortex specimens. Besides, we found that MgSO_4_ ameliorated the overexpression of the Nrg1 and Erbb4 receptors induced by LPS in the hippocampus. Thus, MgSO_4_ treatment for preventing brain injuries can adversely affect offspring cognition behavior later in life, depending on the sex and age of the offspring.

## Graphical abstract

Despite the putative advantages of MgSO_4_ as a neuroprotective agent, the long‐term neuroprotective effect on the offspring is still unraveled. Here, we studied the long‐term impact of antenatal MgSO_4_ at the late gestational periods on the offspring behavior in a mouse MIA model. MgSO_4_ treatment for preventing brain injuries can adversely affect offspring cognition behavior later in life, depending on the sex and age of the offspring.

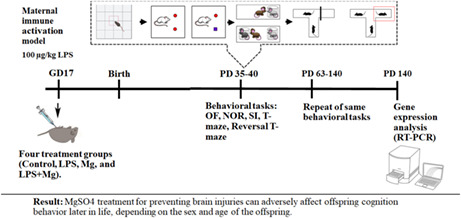

**Significance**

Despite the putative advantages of MgSO_4_ as a neuroprotective agent, the long‐term neuroprotective effect on the offspring is still unraveled. Here, we question the neuroprotection of antenatal MgSO_4_ and provide new information on the yet unknown chain of reactions of the long‐term mechanism and behavior of antenatal MgSO_4_ with and without MIA. Results indicate that female mice are more prone to develop cognitive impairment as a response to the interaction of MIA and MgSO_4_ and pinpoint changes in the expression of cytokines, key components in the ErbB signaling, and the imbalanced expression of dopamine 1 and 2 receptor genes.

## 1 INTRODUCTION

Magnesium is an essential cation in the human body. It is necessary for enzymatic activity, storage, and transfer of energy. Also, considered a calcium antagonist that regulates its availability in the cells. Of note, calcium metabolism and transportation have crucial roles in cardiac function, muscular contraction, blood pressure regulation, and neuronal activity. Furthermore, magnesium has an essential part in endogenous transcellular transports (e.g., physiological blocking of N‐methyl‐D‐aspartate (NMDA) receptor) (Gupta et al., 2014; Shaw & Yager, 2019; Tsakiridis et al., 2020). In clinical practice, it is recommended to administer magnesium sulfate (MgSO_4_) as a neuroprotective agent to women at high risk of imminent preterm birth before 32 weeks of gestation to reduce cerebral palsy (Dowling et al., 2012; Sugimoto et al., 2012; Tam Tam et al., 2011). During the last decade, several prospective studies in pregnant women have demonstrated the neuroprotective effect of MgSO_4_ in preventing preterm white matter brain injury; thereby, MgSO_4_ became “the drug of choice” for decreasing the risk of brain injuries and cerebral palsy in preterm birth, many of whom are associated with chorioamnionitis and maternal immune activation (MIA) exposed fetuses. However, there is no consensus on clear guidelines for MgSO_4_ administration. Few studies discussed the MgSO_4_ potential therapeutic role in the maternal‐fetal axis under inflammation and the long‐term behavior at elementary school age (Daher et al., 2017, 2018; Dowling et al., 2012; Lamhot et al., 2015). On the other hand, clinical studies reported that cognition, motor activity, language ability, and behavior outcomes were not improved by MgSO_4_ therapy (Chollat et al., 2018; Lingam & Robertson, 2018), raising doubts about MgSO_4_ as a treatment of choice for protecting brain development in the long term.

Early immune stimulation induces long‐lasting neuronal changes in animal models, contributing to the emergence of variable neurodevelopmental disorders later in life. Maternal immune activation (MIA) models suggest a relationship between an inflammatory insult (maternal exposure) during pregnancy and the subsequent alteration in offspring behavior and brain neuroanatomy (Izvolskaia & Sharova, 2018). Long‐term behavioral, structural, and functional deficits relevant to schizophrenia and autism spectrum disorder (ASD) are commonly observed in these MIA offspring, including changes in cognitive and social behavior, impaired sensorimotor gating, and increased anxiety, as well as altered cell migration, microglial function, synaptic structure and function (Guma et al., 2019). Furthermore, animal models display additional impairments in working memory, place preference, and novel object recognition (Hemmerke et al., 2017). Our previous study found that adult female offspring from dams exposed to Poly I: C at late gestation displayed a lack of preference for a novel object. In contrast, adult male MIA‐exposed offspring exhibited a working memory alteration. In addition, we report that adult female MIA‐exposed mice spent more time in the center of the open field than female in the control group (Dabbah‐Assadi et al., 2019). Given the cumulating evidence of the role of the ErbB signaling in embryonic brain development (Mei & Nave, 2014), in the biology of the dopaminergic and GABAergic systems (Shamir et al., 2012; Skirzewski et al., 2017; Yan et al., 2018), and schizophrenia pathophysiology (Deng et al., 2013; Mei & Nave, 2014), we investigated the effect of MIA on fetal brain gene expression of these pathways, and the associated neuronal systems (i.e., GABAergic and dopaminergic systems). We demonstrated that 4 h post‐injection of either viral or bacterial mimic material, the expressions of Neuregulin (NRG) 1, its receptor Erbb4, and dopamine D2 receptor (D2R) were altered in the fetal brain (Dabbah‐Assadi et al., 2019). Following that study, we investigated if MgSO_4_ therapy would ameliorate the expression of Nrg1, Erbb4, and D2R and modulate the associated genes, including the ErbB pathway, dopamine, GABA, and glutamate systems (Dabbah‐Assadi et al., 2020). We demonstrated that short‐term treatment with MgSO_4_ affects the expression of Nrg1, Nrg3, dopaminergic markers (e.g., tyrosine hydroxylase (TH), D2R, and D1R), and glutamatergic markers (e.g., GluN1, and GluN2B (subunits for the NMDA receptor)) in the fetal brain. We suggested that these signaling pathways possibly involve the estimated neuromodulation mechanism of MgSO_4_ during gestation (Dabbah‐Assadi et al., 2020). It is unknown whether these short‐term effects of MgSO_4_ maintain during adolescence and adulthood and protect the offspring from developing abnormal behaviors. Here, we explore the long‐term impact of MgSO_4_ administration at the late gestational period on the offspring's behavior in adolescence (1‐month‐old) and adulthood (3‐month‐old). We focused on cognitive function (e.g., working memory, recognition memory), motor activity, and social interaction. At the molecular level, we characterized, in adulthood, changes in gene expression of ErbB signaling and pro‐inflammatory and dopaminergic markers.

## 2 MATERIALS AND METHODS

### 2.1 Animal husbandry

Twenty‐one pregnant female CD1 mice were obtained from Envigo RMS, Inc. (Rehovot, Israel) at Gestation day 11 (GD11, term = 21 days) and housed in the Ort Braude college animal facility, Karmiel, Israel. Animals were treated following NIH Animal Welfare guidelines, and the Ort Braude College Committees approved all the procedures and experiments. All pregnant dams were allowed to acclimate for at least 5 days before the beginning of the study. Pregnant dams were maintained in a temperature‐controlled room on 12 h light/dark cycle (lights on between 7:00 a.m. and 7:00 p.m.) with ad libitum access to food and water. All the behavioral tests were performed during the light phase of the cycle and conducted under dim light illumination.

### 2.2 Maternal immune activation and MgSO_4_ administration

Pregnant mice were randomly assigned into four different treatment groups (*n* = 4 per group): saline/saline (control group), LPS/saline (LPS group), saline/MgSO_4_ (Mg group), and LPS/MgSO_4_ (LPS + Mg group). LPS activation and MgSO_4_ administration were performed as described in Dabbah‐Assadi et al. (2020). At GD17, dams received a single dose of 100 μg/kg LPS (Sigma‐Aldrich, St. Louis, MO) or saline intraperitoneal. Two hours before and 2 h following the LPS or saline injection, dams were treated with MgSO_4_ (270 mg/kg loading dose, followed by 27 mg/kg every 20 minutes: in a subcutaneous route) or saline accordingly. After treatment, pregnant female mice were allowed to deliver spontaneously, and the litters were left undisturbed until weaning. Offspring mice were separated by sex, group‐housed, and maintained as described above. The number of pups per mom is summarized in Table S1.

### 2.3 Behavioral tasks

**FIGURE 1:**
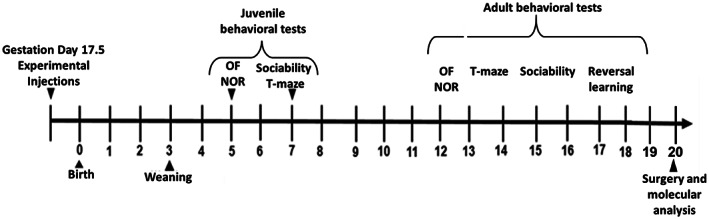
Experiment design and timeline. In brief, at 17GD, pregnant dam mice were injected with saline, LPS, MgSO_4_, or LPS/MgSO_4_. Behavioral tests were performed between PD 35–49 (juvenile) and PD 63–140 (adulthood). At the end of the behavioral tasks, adult offspring's brains were collected for molecular analysis. NOR, novel object recognition; OF, open field test.

Offspring were assigned to behavioral tasks at two developmental periods: in juvenile (postnatal day [PD] 35–49), and the same cohort was re‐tested in adulthood (PD 63–140, Figure 1.) All behavioral studies were performed in the following order: Open field test, Novel Object Recognition test, Social interaction, and T‐maze‐working memory test. T‐maze‐reversal learning was performed only in adulthood. All tests were recorded and designed by the AnyMaze program (Stoelting Co., Wood Dale, IL, USA). The number of animals per task is summarized in Table 1.

**TABLE 1:** A summary of the number of offspring per behavioral task

Task	Saline	LPS	Mg	LPS + Mg
	Young/adult	Young/adult	Young/adult	Young/adult
*Open field*				
Male	23/19	12/12	24/22	10/10
Female	22/17	17/17	21/15	13/13
*NOR*				
Male	23/19	12/12	24/22	10/10
Female	22/17	17/17	21/15	13/13
*Social interaction*				
Male	23/19	12/12	24/22	10/10
Female	22/17	17/17	21/15	13/13
*Working memory*				
Male	23/19	12/12	24/22	10/10
Female	22/17	17/17	21/15	13/13
*Reversal learning* ^a^				
Male	7	7	7	7
Female	7	6	6	7

^a^ Only in adult.

### 2.4 Open field test

The open field test measured locomotor activity in a 40 × 40 cm open field arena. Mice were placed in one corner of the arena, and their movement activity was captured for 30 min with the AnyMaze program. Total locomotor activity and time spent in the chamber center were collected and quantified using the AnyMaze. The open field chamber was wiped between trials with a 70% ethanol solution for odor blurring.

### 2.5 Novel object recognition (NOR) test

The preference of the novelty object was tested as described in Dabbah‐Assadi et al., 2019. The mouse was first habituated to the arena. Then, two identical objects were positioned in the opposite corners of the arena. Each mouse was allowed to explore similar objects for 10 min. Then, the mouse was returned to his home cage for 30 min. In the test phase, one of the objects was replaced by a novel object, and the mouse was allowed to explore the two objects (novel and familiar) for 5 min. The Preference index (PI) was calculated as [(time exploring the novel object)/(time exploring the novel object + time exploring the familiar object)] * 100.

### 2.6 Social interaction test

The sociability test was performed according to Silverman et al. (2010). Each mouse was allowed to explore three empty identical transparent chambers, having two transparent, open doors between them. In phase 1, two identical empty wired cups were placed in the opposite side chamber, and the mouse under test was allowed to explore the whole arenas for 10 min. In the test phase, an unfamiliar CD1 mouse was placed under one of the wired cups. The unfamiliar mouse was of the same sex to avoid sexual attraction behavior. The mouse's time spent in each section was recorded and measured automatically with the AnyMaze program (Stoelting Co., Wood Dale, IL, USA). The social index was calculated as [(time spent in the novel mouse chamber)/(time spent in the novel mouse chamber + empty cup chamber)] * 100.

### 2.7 T‐maze (working memory)

The T‐maze test was performed according to Sarne et al. (2018). The task included force and test phases. In the force phase, the mouse was placed in the centered start arm with the door to one of the goal arms opened (arbitrarily chosen) and allowed to walk freely between the components (start and goal arms) for 7 min. The mouse was removed to its home cage for 5 min before proceeding to the test phase. During the test phase, the two maze doors were open, and the mouse was allowed to re‐explore the maze for 2 min. The number of entries to each arm and the time the mouse spent in the two‐goal arms were recorded and measured automatically with the AnyMaze program. The preference index was calculated as [(time exploring the novel arm)/(time exploring the novel arm + the familiar arm)] * 100.

### 2.8 Reversal T‐maze

T‐maze reversal learning was performed only in adulthood, as described in Golani et al. (2014), with a minor change. The maze was filled in with room‐temperature tap water. The water served as an incentive to reach the platform at the end of one of the arms. Each testing trial contained one force run with one door opened in one arm having a platform (arbitrarily chosen), followed by 10 choice runs with two opened doors. The learning criterion was 80% correct entries for three consecutive days to the selected arm in the forced run (the one with the platform). After learning, the platform's place was switched, and the mice were tested for reversal learning. The number of correct entries and time to reach the learning criterion was scored. Water was replaced in the maze between mice to remove olfactory cues.

### 2.9 Adult brain specimens

At the end of the last behavioral experiment (Postnatal day [PD] 140), offspring mice were sacrificed, prefrontal cortex (PFC) and hippocampus (HC) specimens (*n* = 12 from each treatment, six per sex) were isolated and kept at −80°C until used (for gene expression analysis) (Figure 1).

### 2.10 Gene expression analysis

RNA was extracted from adult HC and PFC samples by combining TRI reagent (Sigma‐Aldrich, St. Louis, MO, USA) and Purelink TM kit (Thermo Fisher Scientific, Waltham, MA, USA). cDNA was prepared, and real‐time PCR was performed as described in detail in Dabbah‐Assadi et al. (2019). RNA quantity and quality were determined using the NanoDrop Spectrophotometers (Thermo Fisher Scientific, Waltham.MA. USA). In brief, according to the manufacturer, 1 μg RNA was converted to cDNA using applied biosystems high‐capacity cDNA Reverse Transcription kit (Thermo Fisher Scientific, Waltham.MA. USA). Each real‐time PCR reaction was in a final volume of 20 μl and included .2 μl of each primer forward and reverse (Table S2), in addition to 10 μl PerfecTa SYBR Green FastMix, ROX (2X), 8 μl DDW, and 2 μl cDNA sample of each reaction. The relative expression of Nrg1, Erbb4, D2DR, D1DR, and IL‐6 normalized to β‐actin and was calculated using the ΔΔCt method.

### 2.11 Statistical analysis

Statistical analysis was conducted using the GraphPad Prism 8.0.2 program and IBM SPSS 25 programs. Changes in exploratory behavior, object recognition, social preference, reversal learning, and preference for the novel arm in young or adult offspring were analyzed by four‐way ANOVA with MIA (Saline or LPS), maternal treatment (saline or Mg), period (adolescence or adulthood), and sex (males or females) as between‐subjects factors, followed by LSD‐corrected t‐test. Statistic results for all the behavioral data are presented in Table S3. Gene expression of adult mice specimens was assessed with three‐way ANOVA with MIA (Saline or LPS), maternal treatment (saline or Mg), and sex (males or females), followed by an LSD‐corrected t‐test. Statistical significance was set at *p* < .05. All data presented as mean ± standard error of the mean (SEM). Of note, unassessed pups were maintained with littermates to avoid additional stress and ensure similar conditions, and equal proportions of mice were utilized per litter to avoid litter effects.

## 3 RESULTS

A summary of the study design is presented in Figure 1.

### 3.1 Long‐Term behavioral effects of MgSO_4_ at juvenile and adulthood

#### 3.1.1 Exploration behavior

**FIGURE 2:**
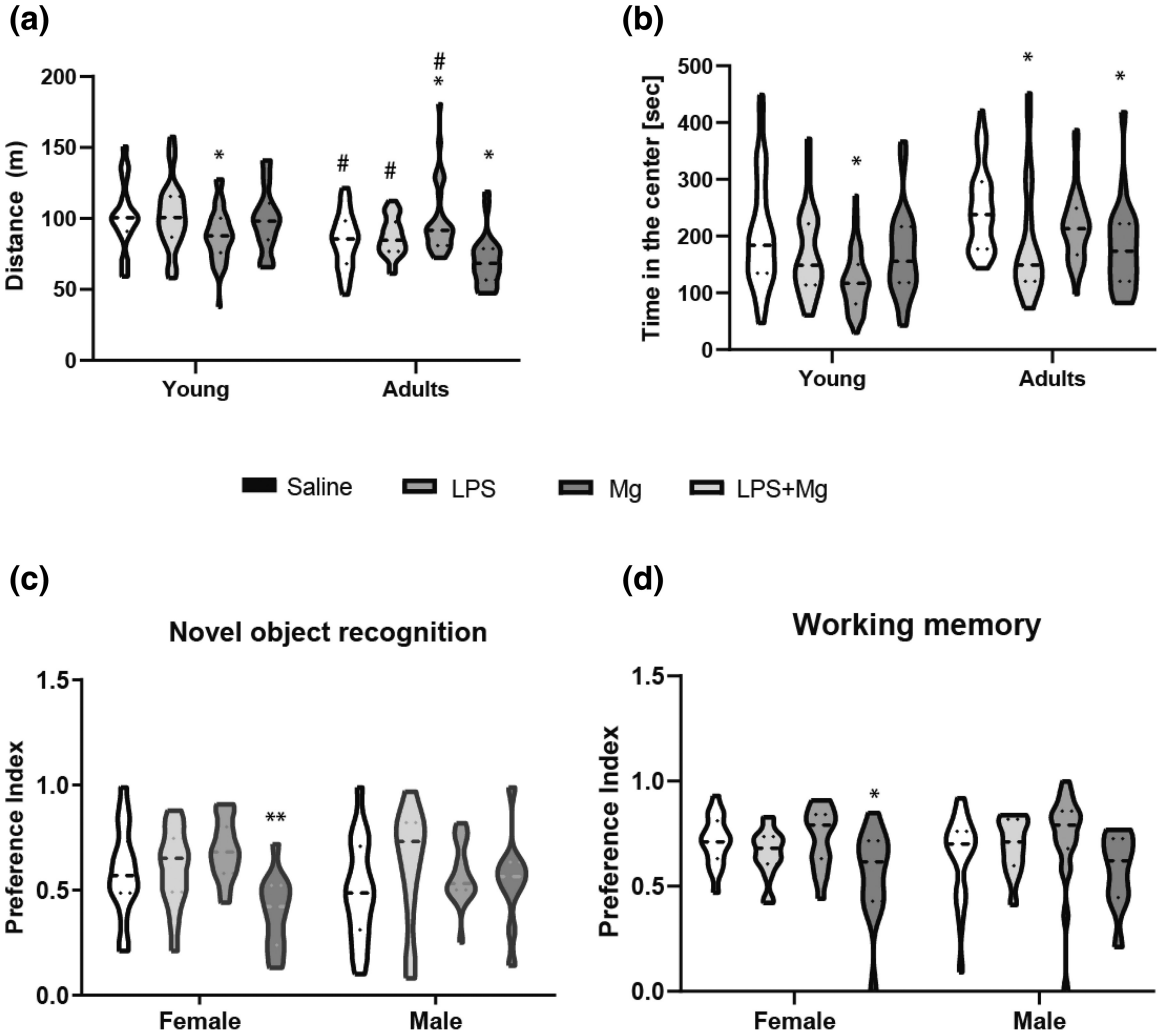
MIA and MgSO_4_ effect on locomotor activity, visual, and working memory. (a) Total distance traveled. (b) Time spent in the center. (c) Novel object test: Adult female LPS + Mg exposed mice did not prefer the novel object over the familiar one. (d) T‐maze—working memory: A reduction in preference for the novel arm was observed in adult females of MgSO_4_ + LPS exposed mice. *n* = 10–24 animals/sex/group (see Table 1). Results are displayed as mean ± SEM. Three‐way ANOVA (a and b), four‐way ANOVA (c and d), followed by LSD‐corrected t‐test. *p < .05; **p < .01 versus saline group. ^#^ *p* < .05 versus LPS + Mg group.

An open field test was carried out to observe exploratory behavior and locomotor activity of the offspring of the antenatal‐treated moms. There was no statistical effect of sex or interaction between sex and maternal treatment (saline or Mg), sex and period (adolescence or adulthood), sex and MIA (saline or LPS), and sex, MIA, maternal treatment, and period (Figure S1). Therefore, both sexes were analyzed together. A significant interaction between MIA, maternal treatment, and period was found (three‐way ANOVA: *F*(1, 264) = 15.3; *p* < .0001). Interaction analysis revealed that young Mg‐treated dam's offspring traveled less in the open arena than in the control group (Fisher LSD: *p* < .005; Figure 2a). Adolescent LPS‐treated dam's offspring traveled the same distance as the control group's offspring (Fisher LSD: *p* > .05). In adulthood, the offspring of Mg‐treated dams presented higher locomotor activity in the open field than in the control group. In contrast, LPS + Mg‐treated dams offspring traveled less distance in comparison to the control group and the other experimental group (Fisher LSD: *p* < .005 vs. control, *p* < .005 vs. LPS and *p* < .0001 vs. Mg; Figure 2a). At the same time, adult LPS‐treated dam's offspring displayed regular locomotor activity in the open field (Fisher LSD: *p* > .05).

Analysis of the time the mouse spent in the center (center time) reveals a significant interaction between MIA and maternal treatment (three‐way ANOVA: *F*(1, 258) = 7.2; *p* < .01; Figure 2b) and between MIA and period (three‐way ANOVA: *F*(1, 258) = 9.2; *p* < .005; Figure 2b). Interaction analysis demonstrated that adolescent offspring from Mg‐treated dams spent significantly less time in the center as compared with the control group and the other comparison groups (Fisher LSD: *p* < .00001 vs. control, *p* < .001 vs. LPS, *p* < .05 vs. LPS + Mg). In adulthood, offspring from LPS and LPS + Mg‐treated dams spent less time in the center than in the control group (Fisher LSD: *p* < .001; Figure 2b).

### 3.2 Novel object recognition and working memory

Analysis of preference for the novel object reveals a significant interaction between maternal treatment, period, and sex [four‐way ANOVA: *F*(3, 258) = 2.70; *p* < .05]. Interaction analysis revealed that in adulthood, but not in adolescence (Figure S2), female offspring, but not male, from dams treated with LPS + Mg did not prefer the novel object over the familiar one as compared with the other treatment groups (Fisher LSD: *p* < .005 vs. control, *p* < .005 vs. LPS, *p* < .00005 vs. Mg; Figure 2c). However, LPS‐treated dams' offspring perform as the control group in the novel object task. In addition, analysis of the working memory task demonstrated a significant effect of the treatment [four‐way ANOVA *F*(3, 258) = 3.86; *p* < .01] with no interaction with any of the subject's factors. Overall, significant working memory impairment was observed in offspring from LPS–Mg‐treated dams (Fisher LSD: *p* < .005 vs. control, *p* < .005 vs. LPS, *p* < .005 vs. Mg; Figure 2c). Within‐group analysis of adult mice revealed that females, but not males, from the LPS + Mg‐treated moms spent less time in the novel arm than in the saline‐treated group and the LPS‐treated group [*F*(3,116) = 4.01; *p* < .001, Figure 2d] as measured by the preference index. No significant change in working memory was observed in adolescence (Figure S2).

#### 3.2.1 T‐Water maze reversal learning and social interaction

**FIGURE 3:**
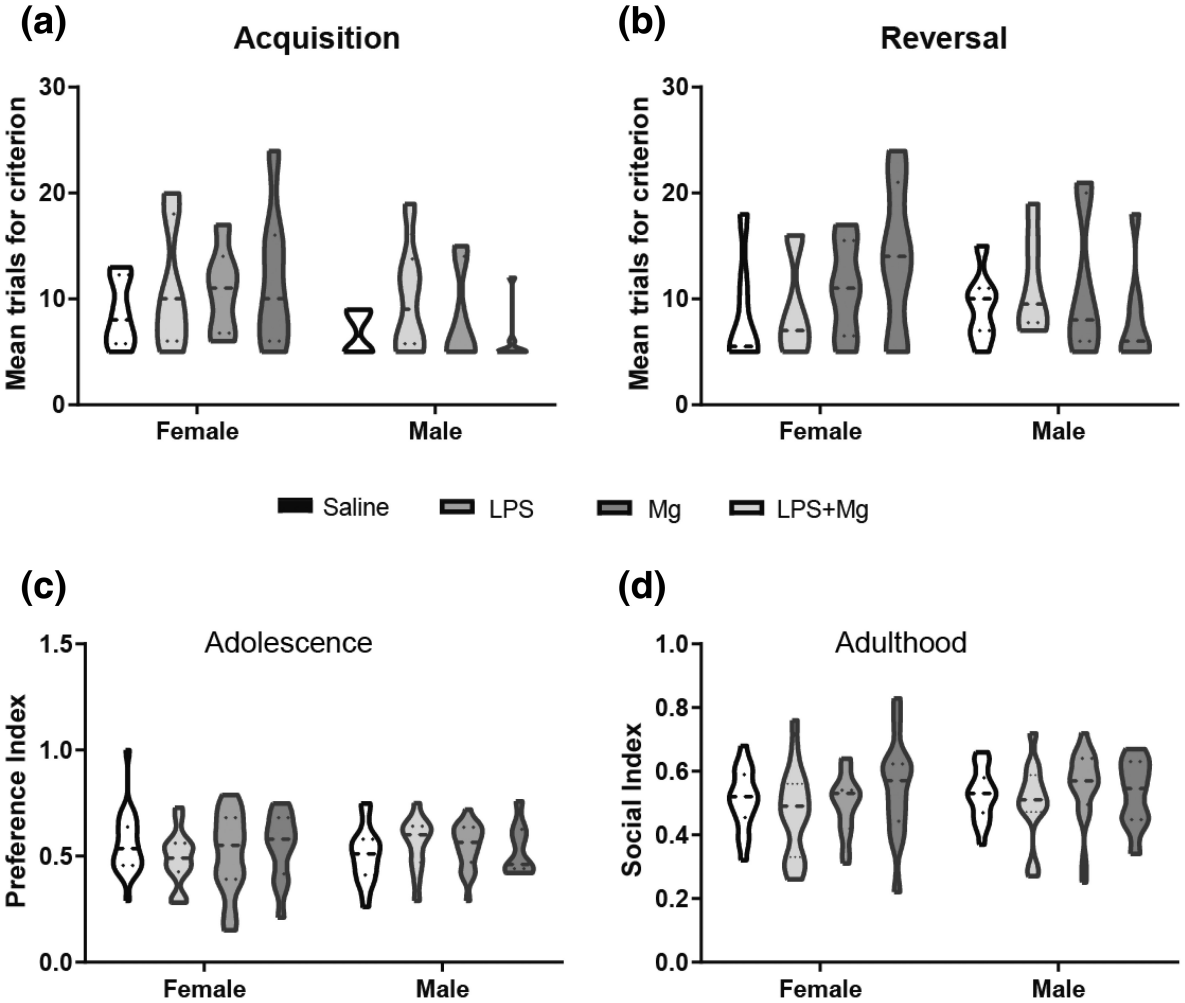
MIA and MgSO_4_ effect on reversal learning and social interaction. (a) Represents the acquisition phase of the reversal learning task. (b) Represents the reversal phase of the reversal learning task (c) represents the social index of the social interaction test in adolescence. (d) Represents the social index of the social interaction test in adulthood. *n* = 7–22 animals/sex/group (see Table 1). Four‐way ANOVA followed by LSD‐corrected *t*‐test. Results are displayed as mean ± SEM.

In the T‐maze reversal learning, the mouse first distinguishes between the arm with a platform and without one. Then, the mice learn to reverse their choice. Overall, male offspring reached the learning criterion (80% correct entries for three consecutive days) in fewer days than female offspring (three‐way ANOVA: *F*(1, 52) = 4.64; *p* < .05). However, there was no significant difference between the experimental groups in reaching the learning criterion during the conditional discrimination learning (acquisition phase, Figure 3a) and the reversal learning phase (Figure 3b). Social preference index data also reveal no significant main effect of MIA, maternal treatment, sex, and period (*p* > .05). Thus, no social interaction impairment was observed in adolescence or adulthood (Figure 3c,d, respectively).

#### 3.2.2 Long‐Term molecular changes of MgSO_4_ administration

**FIGURE 4:**
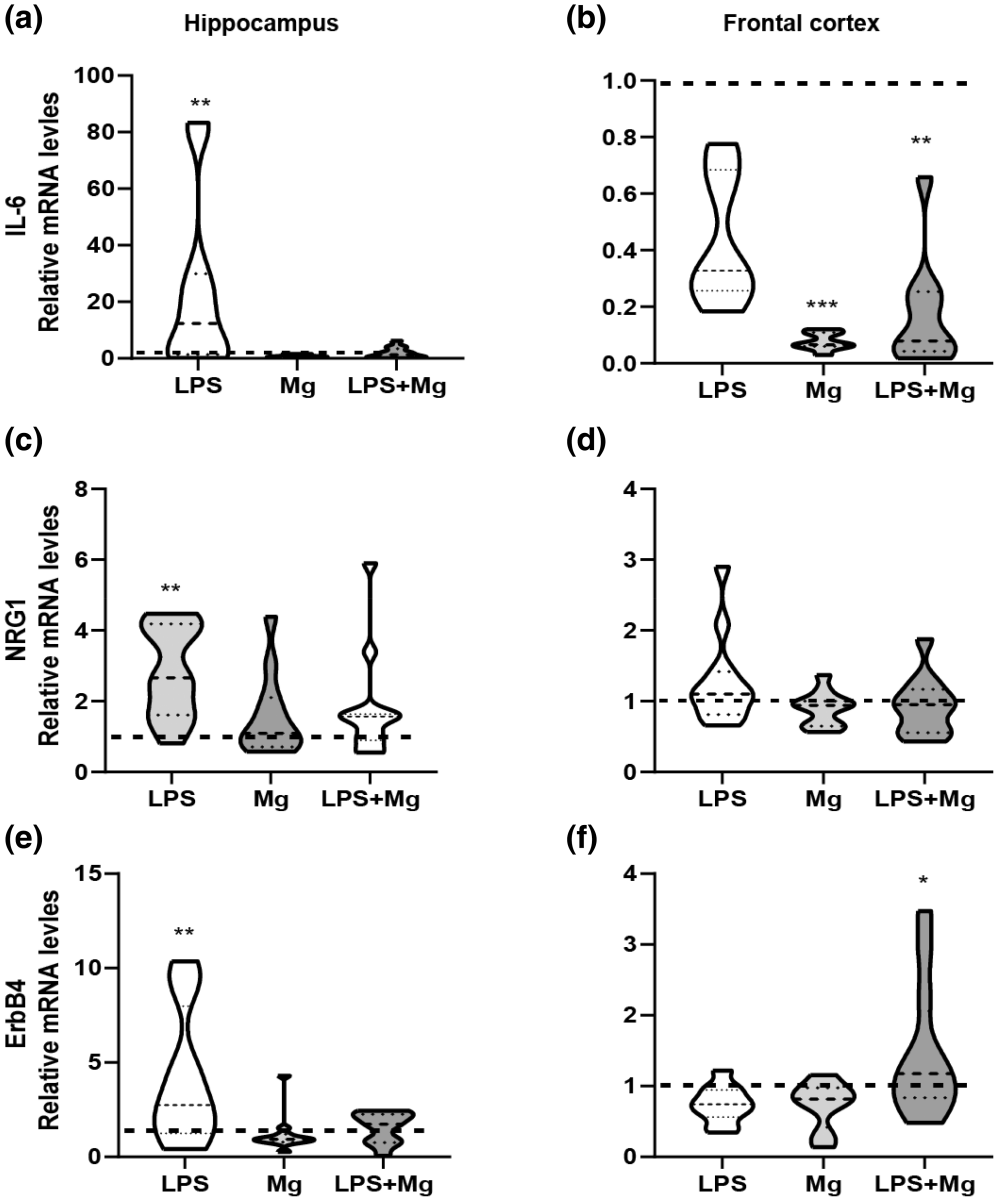
Effect of MIA and MgSO_4_ administration on IL6, Nrg1, and ErbB4 expression in the adult hippocampus and frontal cortex. IL‐6 expression; analysis showed a significant elevation was observed in offspring from the LPS‐treated dams in the hippocampus (a), and reduction in the prefrontal cortex (PFC) of offspring from Mg‐treated dams (b). Nrg1 expression; analysis showed a significant elevation in the hippocampus of offspring from LPS‐treated dams (c) but not in the PFC (d). Erbb4 expression; analysis showed a significant elevation in the hippocampus of offspring from LPS‐treated dams (e) and the PFC of the LPS + Mg group (f). Saline, black dashed line. Results are displayed as mean ± SEM. *n* = 12 in each group. One‐way ANOVA followed by LSD‐corrected *t*‐test. *p < .05; **p < .01; ***p < .001.

PFC and hippocampus expression levels of Nrg1, its receptor Erbb4, inflammatory cytokine IL‐6, and dopamine receptors 1 and 2 of adult males and females were evaluated. No effect of sex or interaction between MIA, sex, and treatment was observed. Therefore, both sexes were analyzed together. A significant elevation of Il6 was observed in offspring from the LPS‐treated dams in the hippocampus as compared to the other comparison groups, which was alleviated by MgSO_4_ [*F*(3, 44) = 6.23; *p* < .001, Figure 4a]. A significant reduction of the Il6 expression levels was also observed in the PFC of offspring from Mg‐treated dams as compared with the saline and the LPS‐treated groups [*F*(3, 30) = 9.94; *p* < .0001, Figure 4b].

An elevation of Nrg1 expression [*F*(3, 44) = 6.23; *p* < .005; Figure 4c] and its receptor Erbb4 was found in the hippocampus of offspring from LPS‐treated dams, [*F*(3, 42) = 6.93; *p* < .001; Figure 4e]. Treatment with MgSO_4_ was sufficient to alleviate the expression to the control level. In the PFC, a significant elevation of Erbb4 was observed in offspring of the Mg–LPS‐treated group but not of the MgSO_4_ or LPS‐treated group as compared with the control group [*F*(3, 38) = 4.06; *p* < .01, Figure 4f]. No difference in Nrg1 expression was observed in the PFC (Figure 4d).

**FIGURE 5:**
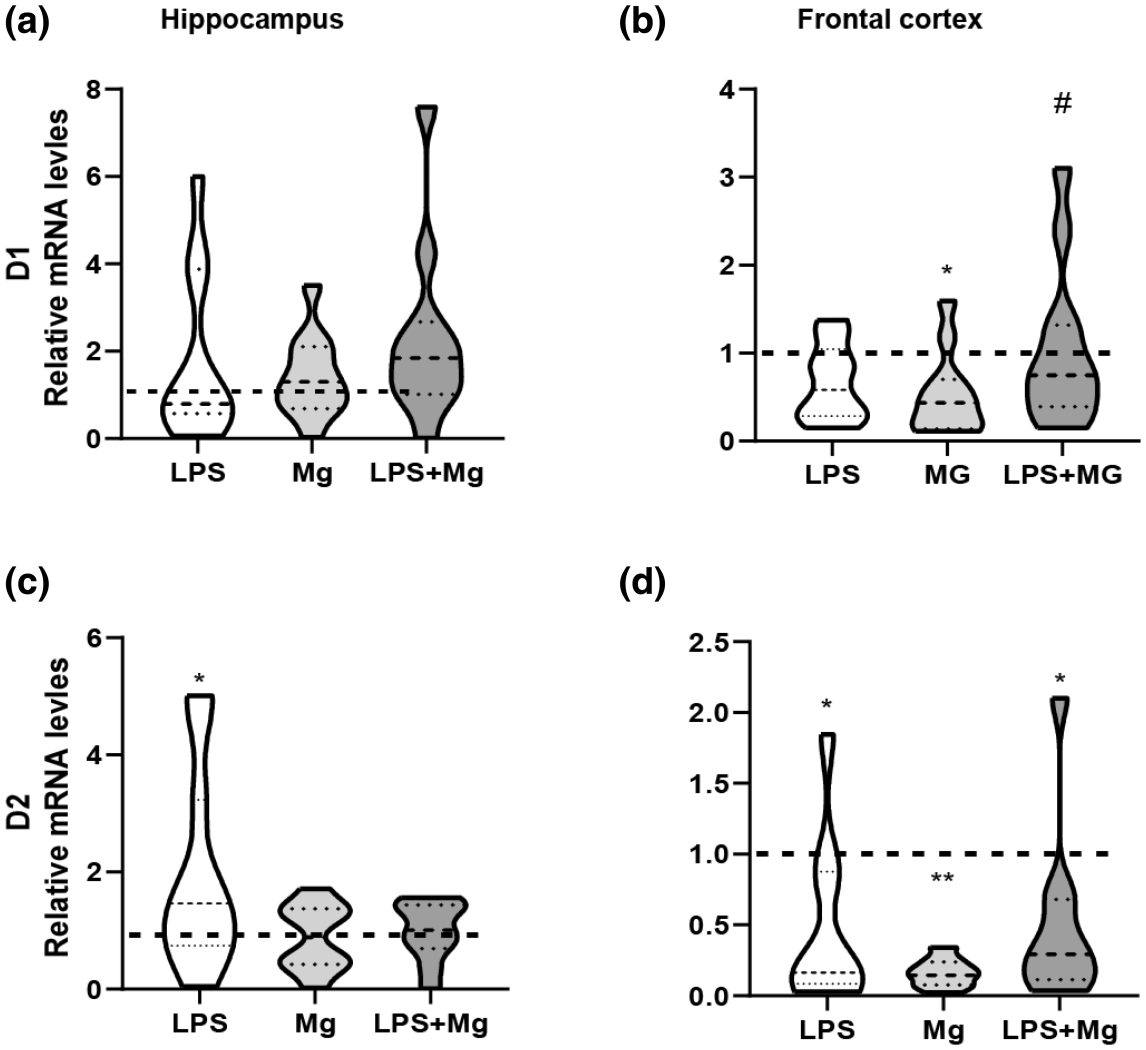
Effect of MIA and MgSO_4_ administration on D1R and D2R in the adult hippocampus and PFC. D1R expression; No difference in the expression levels in the hippocampus (a), while a significant downregulated in offspring from the Mg‐treated dams was observed (b). DR2 expression; analysis showed a significant elevation in the hippocampus of offspring from LPS‐treated dams (c), and reduced levels were demonstrated in all the treatment groups (d). Saline, black dashed line. Results are displayed as mean ± SEM. *n* = 12 in each group. One‐way ANOVA followed by LSD‐corrected t‐test. *p < .05; **p < .01; ^#^ *p* < .05 versus Mg group.

As shown in Figure 5, in the PFC D1DR, mRNA expression was significantly downregulated [*F*(3, 44) = 5.09; *p* < .005] in offspring from the Mg‐treated dams. At the same time, a significant reduction of D2DR receptors in all the treatment groups was found as compared to the control group [*F*(3, 38) = 7.11; *p* < .001]. In the hippocampus, a significant elevation of D2DR expression was observed in the offspring of the LPS‐treated groups [*F*(3, 40) = 3.16; *p* < .05], with no significant changes in the expression of D1DR.

## 4 DISCUSSION

Antenatal prophylactic magnesium sulfate in pregnant women is a needed clinical intervention that manifests neuroprotective and anti‐inflammatory effects to reduce cerebral palsy cases and long‐term pregnancy complications. However, the long‐term outcomes of MgSO_4_ are controversial and remain unclear. Here, we evaluated the long‐term outcomes associated with prophylactic MgSO_4_. Compared with the control group, offspring from MgSO_4_‐treated dams demonstrate a significant abnormal exploration behavior in adolescence and adulthood. We further show that MgSO_4_ administration and activation of the maternal immune system resulted in offspring cognitive performance, which was dependent on the sex of the mouse. Nevertheless, adult offspring from the LPS–Mg‐treated group presented hypo‐locomotor activity and spent less time in the open field center, similar to the LPS‐treated group. A reduction in IL‐6 expression was observed in the PFC and hippocampus of the LPS–Mg‐treated groups. At the same time, an imbalance expression of the dopamine D1 and D2 expression was observed only in the PFC. Besides, we found that MgSO_4_ ameliorated the Nrg1, ErbB4, and D2 receptor gene expression in the hippocampus. Our findings provide new information on the yet unknown chain of reactions of the long‐term molecular impact and behavior of antenatal MgSO_4_ exposed offspring with and without MIA.

Here we report a significant alteration in locomotor activity of MgSO_4_‐treated dams. We observed a reduction in total motor activity during adolescence and the time the mice spent in the center of the open field arena. The last observation implies higher anxiety. Contrarily, in adulthood, we found that the offspring from the MgSO_4_‐treated dams were hyperactive and spent the same amount of time in the center of the open field. These data suggest that the observed changes depend on the mice's age, implying different “MgSO_4_ phenotypes” throughout development. Furthermore, adult offspring from the LPS–Mg‐treated group presented hypo‐locomotor activity and, like the LPS‐treated group, spent less time in the center of the arena, suggesting that in adulthood, antenatal MgSO_4_ did not protect the offspring from developing anxiety‐like behavior induced by triggering the maternal immune system at late gestational day. We further demonstrate that MgSO_4_ administration and activation of the maternal immune system at the late stage of pregnancy resulted in offspring cognitive dysfunction. Specifically, adult females exposed to MgSO_4_ under inflammation preferred to spend less time in the novel arm and next to the unknown object, suggesting sex differences in working and recognition memory function. Thus, our data indicate that females are more prone to develop cognitive impairment as a response to the interaction of MIA with MgSO_4_, suggesting that there is a cumulative effect of LPS and MgSO_4_ that is detrimental. It is essential to emphasize that the observed cognitive impairment is only relative to the control group, and LPS‐induced MIA did not alter cognitive performance, as we previously reported (Dabbah‐Assadi et al., 2019). However, although MIA did not have a lasting effect on reversal learning and recognition memory, we can question the neuroprotection of antenatal MgSO_4_ as a response to maternal inflammation since antenatal MgSO_4_ did not alter cognitive performance in female mice. Interestingly, in a pregnancy complication such as preeclampsia, prophylactic MgSO_4_ did not protect the offspring from long‐term cognition and behavioral difficulty at school age. It has been reported that preeclampsia offspring (7–10 years), who underwent psychometric testing, developed a working memory impairment (Rätsep et al., 2016). Nevertheless, these results do not align with previous preclinical studies that reported MgSO_4_ pretreatment benefits in the preference of the novel object in females with cerebral damage (Daher et al., 2018) or ameliorated the learning and memory impairment of MIA‐induced offspring in adulthood (Lamhot et al., 2015). Galinsky and colleagues (2017) reported that MgSO_4_ administration had anticonvulsant effects in preterm fetal sheep after asphyxia. However, they presented controversial results that MgSO_4_ did not reduce the asphyxia‐induced brain injury and the exacerbated loss of oligodendrocytes followed by asphyxiation. Together, the results question the long‐term neuroprotection effect of MgSO_4_ and suggest that the antenatal magnesium sulfate effect is dependent on the mouse's age and sex and could adversely affect the offspring's behavior later in life.

MgSO_4_ provides neuroprotection by preserving cellular energy metabolism and reducing the inflammatory response. Moreover, preterm parturition may be associated with a fetal inflammatory response syndrome defined by increased Il6 levels and other inflammatory cytokines. We recently demonstrated that MIA at late gestation altered the expression of Nrg1, its receptor Erbb4, and the dopamine D2 receptor 4 h post‐injection of viral or bacterial mimic material in the fetal brain (Dabbah‐Assadi et al., 2019). We detected a significant downregulation of Il‐6 expression in the hippocampus and PFC of adult MgSO_4_‐treated MIA offspring. Furthermore, we found that MgSO_4_ significantly ameliorated Nrg1 and Erbb4 levels in the hippocampus but not in the PFC. Our current and previous published data (Dabbah‐Assadi et al., 2019) suggest that antenatal MgSO_4_ administration probably exerts its neuroprotection which might be reflected in normal social behavior and reversal learning by downregulating the expression of inflammatory cytokine and reducing the expression of key components of the ErbB signaling activation in a region‐specific manner. Further research is needed to study whether the observed results is due to downregulation of cytokine production and NRG–ErbB4 pathway activity. We also suggest that antenatal MgSO_4_ administration plays a critical role in cytokine gene expression throughout brain development. Besides, we demonstrated for the first time a reduction in D2DR expression and elevation of D1DR in the PFC, with no change in the hippocampus, in antenatal magnesium sulfate administrated offspring under inflammatory conditions. Dopamine in the prefrontal cortex plays an essential role in working memory. For example, enhancing D2DR activity in the PFC in monkeys increases the persistent working memory responses (Ott & Nieder, 2017). Pretreatment with a D1DR antagonist attenuated dose‐related spatial working memory impairments induced by intra‐PFC injections of D1DR receptor agonists (Kristofova et al., 2017). Thus, the imbalance of dopamine receptor expression in the PFC of antenatal magnesium sulfate administration during pregnancy in adulthood could be involved in the molecular mechanism of sex‐dependent memory impairment. Further studies are needed to explore this possibility.

Studying the molecular neuroprotection mechanism of MgSO_4_ is not feasible in humans. Therefore, modeling a specific aspect of fetal brain injury in animals can serve as a good platform for this study. Thus, using an animal model is the strength and limitation of this study. Another limitation of the study is that preterm birth MgSO_4_ treatment in humans is not always associated with MIA. Therefore, we cannot conclude about other cases of prophylactic MgSO_4_, although our data suggest that MgSO_4_ by itself may affect behavior later in life. In addition, we investigated the neuroprotective effect of MgSO_4_ and MIA at the mRNA expression level and not the protein levels. Future studies are needed to explore or support the MgSO_4_ effect on long‐term gene expression that we assume is also reflected at the protein level. Furthermore, the impact of MIA on behavioral phenotype depends on the time in which the maternal immune system was activated. In previous publications we observe different MIA protocols which might explain the inconsistency in findings. For example, it was reported that MIA activation in the mid‐late gestational (GD15) day led to alterations in social performance (Fernández de Cossío et al., 2017), while on GD17, it led to hyper locomotor activity (Careaga et al., 2017). Also on GD12, but not GD16, MIA resulted in reward‐seeking behavior alteration (Straley et al., 2016). Thus, our findings correspond with the GD17 MIA impact. Finally, yet importantly, in the current study, the prophylactic dose and the administration of MgSO_4_ follow the literature and previous work (Dabbah‐Assadi et al., 2019, 2020; Lamhot et al., 2015). While in humans, the doses of antenatal MgSO_4_ are diverse, starting from total treatments of an average of 4 g per patient to 30 g (ACOG, 2010), depending on the protocol used. Thus, diverse protocols and doses will probably affect the offspring outcomes differently.

We conclude that the long‐term outcome of antenatal MgSO_4_, used in the last two decades as neuroprotection in various preterm conditions with no clear guidelines, on locomotor activity depends on age, with a different phenotype in adolescence and adulthood. In addition, we suggest the MgSO_4_ neuroprotection on cognitive function is sex dependent. Further studies focusing on sex and the time the maternal immune system was activated are needed to shed more light on the long‐term molecular mechanism and behavior of MIA and antenatal magnesium sulfate administration during pregnancy.

### DECLARATION OF TRANSPARENCY

The authors, reviewers and editors affirm that in accordance to the policies set by the *Journal of Neuroscience Research*, this manuscript presents an accurate and transparent account of the study being reported and that all critical details describing the methods and results are present.

## AUTHOR CONTRIBUTIONS

A.S. and I.G. designed the current project. F.D‐A. performed the experiments. S.R. assisted with the behavioral experiments. A.R. assisted with the molecular experiments. A.S. and F.D‐A. analyzed the data. A.S. and F.D‐A. wrote the paper. R.D., D.A., and E.P. assisted with the interpretation of the results and the discussion. All the authors have contributed to and approved the final manuscript for publication.

## FUNDING INFORMATION

The National Institute of Psychobiology in Israel (231‐19‐20, A.S), the Medical leadership program in the Galilee, Galilee developmental Authority (D.A and A.S), and Ort Braude College (I.G.).

## CONFLICT OF INTEREST

The authors declare no conflict of interest.

### PEER REVIEW

The peer review history for this article is available at https://publons.com/publon/10.1002/jnr.25148.

## Supporting information


**FIGURE S1** Effect of MIA and MgSO_4_ on locomotor activity per sex: (a–c) Total distance traveled. (b–d) Time spent in the center. *n* = 10–24 animals/sex/group. Results are displayed as mean ± SEM
**FIGURE S2** Effect of MIA and MgSO_4_ on visual and working memory in adolescence: (a) Novel object recognition (b) Working memory. *n* = 10–24 animals/sex/group. Results are displayed as mean ± SEM

**TABLE S1** Numbers of pups/offspring per mice treatment
**TABLE S2** Primers sequences
**TABLE S3** Statistical results for all behavioral data
**TABLE S4** Results summary of gene expression in the hippocampus and prefrontal cortex

Transparent Science Questionnaire for Authors

## Data Availability

The data supporting this study's findings are available on request from the corresponding authors.
