# Supplementary material for: Long‐term effects of prophylactic MgSO4 in maternal immune activation rodent model at adolescence and adulthood
Source: J Neurosci Res. 2022 Nov 26;101(3):316–26. doi: 10.1002/jnr.25148 (PMC10100175; doi:10.1002/jnr.25148)
Supplement: Supplementary file 1 — FIGURE S1 Effect of MIA and MgSO4 on locomotor activity per sex: (a–c) Total distance traveled. (b–d) Time spent in the center. n = 10–24 animals/sex/group. Results are displayed as mean ± SEM FIGURE S2 Effect of MIA and MgSO4 on visual and working memory in adolescence: (a) Novel object recognition (b) Working memory. n = 10–24 animals/sex/group. Results are displayed as mean ± SEM [file JNR-101-316-s001.docx]

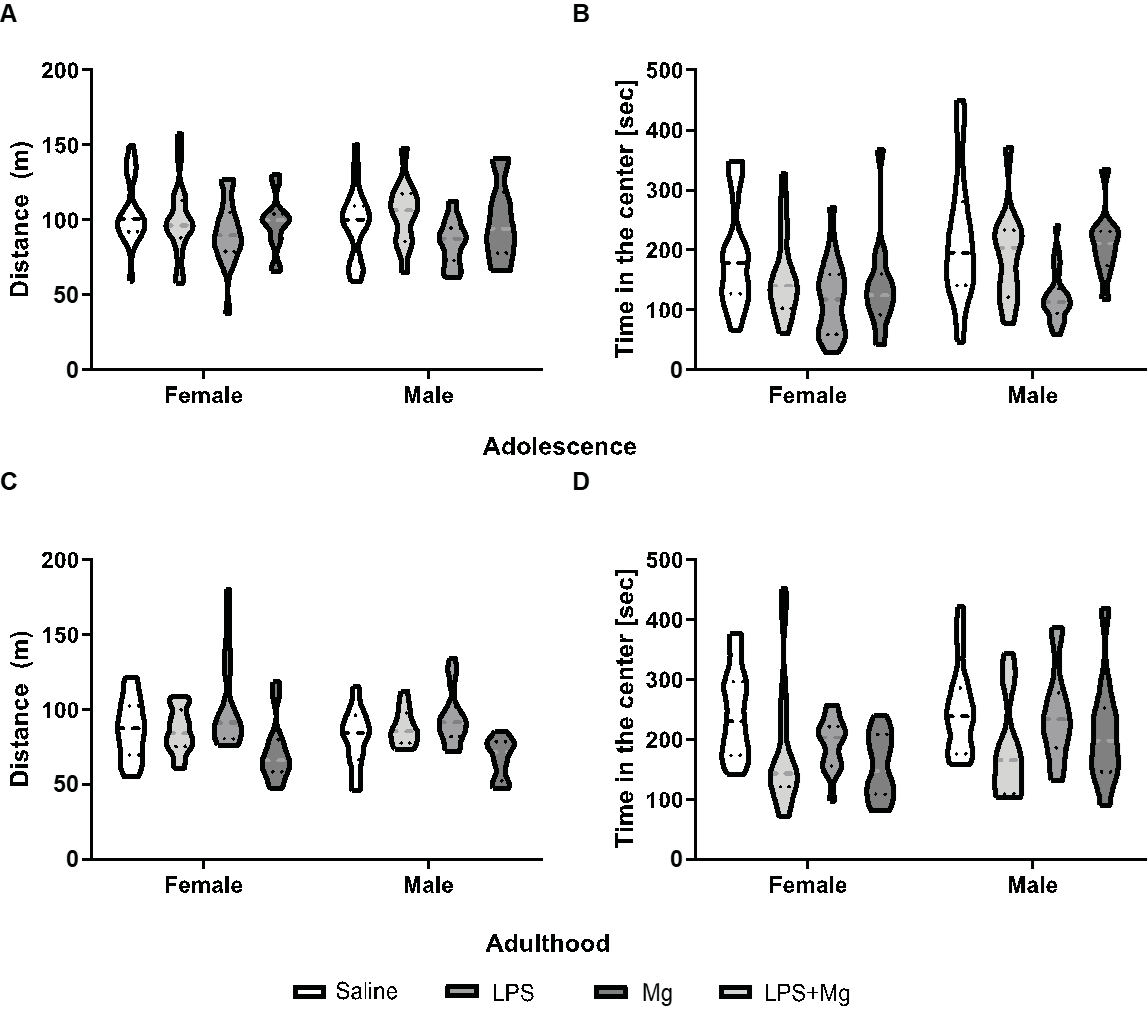


**Supplementary Figure 1. Effect of MIA and MgSO4 on locomotor activity per sex**: A-C) Total distance traveled. B-D) Time spent in the center. n=10-24 animals/sex/group. Results are displayed as mean ± SEM


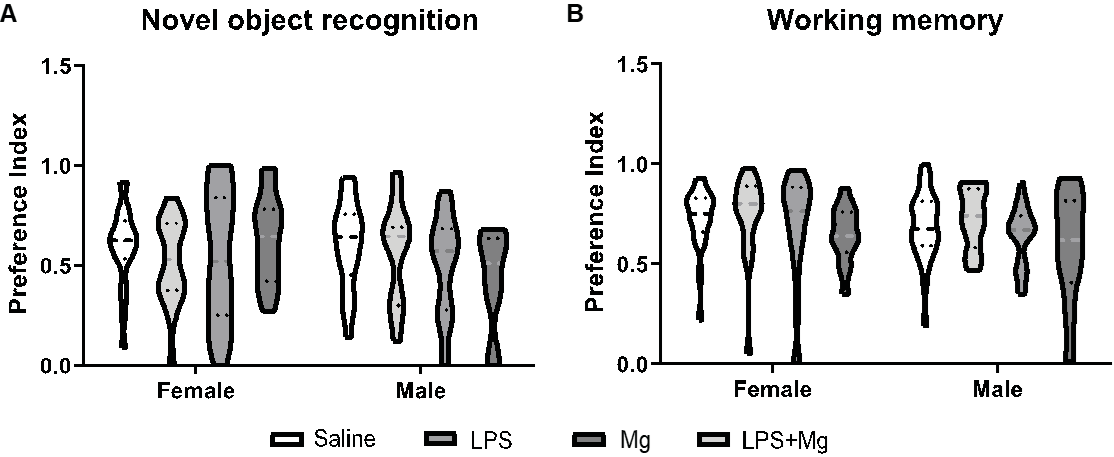


**Supplementary Figure 2. Effect of MIA and MgSO4 on visual and working memory in adolescence**: A) Novel object recognition B) Working memory. n=10-24 animals/sex/group. Results are displayed as mean ± SEM
