# Supplementary material for: Long‐term effects of prophylactic MgSO4 in maternal immune activation rodent model at adolescence and adulthood
Source: J Neurosci Res. 2022 Nov 26;101(3):316–26. doi: 10.1002/jnr.25148 (PMC10100175; doi:10.1002/jnr.25148)
Supplement: Supplementary file 2 — TABLE S1 Numbers of pups/offspring per mice treatment TABLE S2 Primers sequences TABLE S3 Statistical results for all behavioral data TABLE S4 Results summary of gene expression in the hippocampus and prefrontal cortex [file JNR-101-316-s002.docx]

**Supplementary table 1: Numbers of pups/offspring per mice treatment**

| **Total** | **Females** | **Males** | **Group name** |
| --- | --- | --- | --- |
| 11 | 5 | 6 | Saline |
| 12 | 6 | 6 | Saline |
| 14 | 6 | 8 | Saline |
| 9 | 6 | 3 | Saline |
| **46** | **23** | **23** | **Total** |
| 6 | 3 | 3 | LPS |
| 9 | 6 | 3 | LPS |
| 6 | 5 | 1 | LPS |
| 8 | 3 | 5 | LPS |
| **29** | **17** | **12** | **Total** |
| 11 | 7 | 4 | Mg |
| 14 | 8 | 6 | Mg |
| 11 | 6 | 5 | Mg |
| 15 | 6 | 9 | Mg |
| **51** | **27** | **24** | **Total** |
| 8 | 3 | 5 | LPS+Mg |
| 7 | 5 | 2 | LPS+Mg |
| 8 | 5 | 3 | LPS+Mg |
| 8 | 3 | 5 | LPS+Mg |
| **31** | **16** | **15** | **Total** |

**Supplementary table 2: Primers sequences**

|  | | **Primer sequences** |
| --- | --- | --- |
| **ErbB4** | Forward | AGTGGTCTGTCATTGCTTATCCTC |
|  | Reverse | CTGTTGTCCGTGATGTAGATATTGC |
| **Nrg1** | Forward | ACCAGCCATCTCATAAAGTGCG |
|  | Reverse | TTGACGGGTTTGACAGCTCC |
| **IL-6** | Forward | TCCAGTTGCCTTCTTGGGAC |
|  | Reverse | GTGTAATTAAGCC'TCCGACTTG |
| **D1DR** | Forward | TTCTTCCTGGTATGGGTTGG |
|  | Reverse | AGGACAGGACCCAGACAATG |
| **D2DR** | Forward | TATGCCCTGGGTCGTCTATC |
|  | Reverse | AGGACAGGACCCAGACAATG |

**Supplementary table 3: Statistical results for all behavioral data**

|  | Variables | Df | SS | MS | F | P-value | |
| --- | --- | --- | --- | --- | --- | --- | --- |
| **Open Field** |  |  |  |  |  |  | |
| Total distance | Treatment (Saline/MIA/MgSO4) | 3 | 652.285 | 1224.507 | 2.845 | **.038** | |
|  | Sex (Male/Female) | 1 | 537.480 | 520.148 | 1.208 | .273 | |
|  | Period  Adolescent/ adulthood | 1 | 1318.915 | 10062.895 | 23.377 | **.00002** | |
|  | Treatment x Sex | 3 | 40.023 | 384.371 | .893 | .445 | |
|  | Treatment x Period | 3 | 589.986 | 4475.591 | 10.397 | **.00002** | |
|  | Sex x Period | 1 | 45.385 | 30.476 | .071 | .790 | |
|  | Treatment x Sex x Period | 3 | 10.720 | 42.497 | .099 | .961 | |
|  | Residual | 249 | 107187.038 | 430.470 |  |  | |
|  | Total | 265 | 2370673.118 |  |  |  | |
| Center time | Treatment (Saline/MIA/MgSO4) | 3 | 151061.708 | 50353.903 | 8.574 | **.00001** | |
|  | Sex (Male/Female) | 1 | 53457.646 | 53457.646 | 9.102 | **.003** | |
|  | Period  Adolescent/ adulthood | 1 | 93572.927 | 93572.927 | 15.933 | **.00001** | |
|  | Treatment x Sex | 3 | 20201.754 | 6733.918 | 1.147 | .331 | |
|  | Treatment x Period | 3 | 76768.909 | 25589.636 | 4.357 | **.005** | |
|  | Sex x Period | 1 | 930.371 | 930.371 | .158 | .691 | |
|  | Treatment x Sex x Period | 3 | 13719.467 | 4573.156 | .779 | .507 | |
|  | Residual | 250 | 1468252.515 | 5873.010 |  |  | |
|  | Total | 266 | 11220257.340 |  |  |  | |
| **NOR** | Treatment (Saline/MIA/MgSO4) | 3 | .219 | .073 | 1.420 | .238 | |
|  | Sex (Male/Female) | 1 | .033 | .033 | .644 | .423 | |
|  | Period  Adolescent/ adulthood | 1 | .006 | .006 | .125 | .724 | |
|  | Treatment x Sex | 3 | .131 | .044 | .847 | .469 | |
|  | Treatment x Period | 3 | .395 | .132 | 2.565 | .055 | |
|  | Sex x Period | 1 | .011 | .011 | .221 | .639 | |
|  | Treatment x Sex x Period | 3 | .422 | .141 | 2.736 | **.044** | |
|  | Residual | 246 | 12.634 | .051 |  |  | |
|  | Total | 262 | 96.156 |  |  |  | |
| **WM** | Treatment (Saline/MIA/MgSO4) | 3 | .415 | .138 | 3.865 | **.010** | |
|  | Sex (Male/Female) | 1 | .028 | .028 | .783 | .377 | |
|  | Period  Adolescent/ adulthood | 1 | .014 | .014 | .381 | .538 | |
|  | Treatment x Sex | 3 | .032 | .011 | .295 | .829 | |
|  | Treatment x Period | 3 | .180 | .060 | 1.677 | .172 | |
|  | Sex x Period | 1 | .016 | .016 | .459 | .499 | |
|  | Treatment x Sex x Period | 3 | .032 | .011 | .297 | .828 | |
|  | Residual | 251 | 8.994 | .036 |  |  | |
|  | Total | 267 | 132.003 |  |  |  | |
| **SI** | Treatment (Saline/MIA/MgSO4) | 3 | .020 | .007 | .412 | .744 |  |
|  | Sex (Male/Female) | 1 | .050 | .050 | 3.057 | .082 |  |
|  | Period  Adolescent/ adulthood | 1 | .025 | .025 | 1.512 | .220 |  |
|  | Treatment x Sex | 3 | .055 | .018 | 1.115 | .343 |  |
|  | Treatment x Period | 3 | .011 | .004 | .233 | .874 |  |
|  | Sex x Period | 1 | .023 | .023 | 1.403 | .237 |  |
|  | Treatment x Sex x Period | 3 | .040 | .013 | .807 | .491 |  |
|  | Residual | 246 | 4.077 | .016 |  |  |  |
|  | Total | 262 | 75.735 |  |  |  |  |
| **RR**  Acquisition | Treatment (Saline/MIA/MgSO4) | 3 | 46.362 | 15.454 | .722 | .545 |  |
|  | Sex (Male/Female) | 1 | 99.430 | 99.430 | 4.642 | **.037** |  |
|  | Treatment x Sex | 3 | 44.728 | 14.909 | .696 | .559 |  |
|  | Residual | 44 | 942.429 | 21.419 |  |  |  |
|  | Total | 52 | 5515.000 |  |  |  |  |
| **RR**  Reversal | Treatment (Saline/MIA/MgSO4) | 3 | 40.976 | 13.659 | .483 | .696 |  |
|  | Sex (Male/Female) | 1 | 5.752 | 5.752 | .203 | .654 |  |
|  | Treatment x Sex | 3 | 110.319 | 36.773 | 1.300 | .286 |  |
|  | Residual | 45 | 1273.024 | 28.289 |  |  |  |
|  | Total | 52 | 7058.000 |  |  |  |  |

Three Way ANOVA, LSD-corrected t-test

SS=Sum of squares; MS=mean squaresMIA= Maternal immune activation; NOR= Novel Object Recognition; WM=Working memory; RR=Reversal learning; SI=Social interaction.

**Supplementary Table 4: Results summary of gene expression in the hippocampus and prefrontal cortex**

|  | **Hippocampus** | | | | **Prefrontal Cortex** | | | |
| --- | --- | --- | --- | --- | --- | --- | --- | --- |
|  | **LPS** | **Mg** | **LPS+ Mg** | **P-value** | **LPS** | **Mg** | **LPS+ Mg** | **P-value** |
| **IL-6** | **22.83±8.57** | 0.77±0.13 | 1.99±0.57 | p<0.001 | 0.43±0.08 | **0.07±0.01** | **017±0.06** | p<0.0001 |
| **Nrg1** | **2.83±0.37** | 1.58±0.33 | 1.79±0.43 | p<0.005 | 1.27±0.18 | 0.89±0.07 | 0.93±0.12 | p>0.05 |
| **ErbB4** | **4.17±1.02** | 1.23±0.32 | 1.56±1.02 | p<0.001 | 0.74±0.08 | 0.73±0.12 | **1.45±0.27** | p<0.01 |
| **D2DR** | **1.92±1.69** | 0.88±0.56 | 0.99±0.50 | p<0.05 | **0.47±0.61** | **0.16±0.10** | **0.48±0.61** | p<0.001 |
| **D1DR** | 1.77±1.94 | 1.41±0.95 | 2.28±2.07 | p>0.05 | 0.73±0.45 | **0.57±0.54** | **1.1±0.81** | p<0.005 |

Results are presented in arbitrary units ±SEM and normalized to β-actin gene expression and the saline group. The bold results represent the statistically significant values. One-way ANOVA, LSD-corrected t-test
