# Supplementary material for: Long‐term effects of prophylactic MgSO4 in maternal immune activation rodent model at adolescence and adulthood
Source: J Neurosci Res. 2022 Nov 26;101(3):316–26. doi: 10.1002/jnr.25148 (PMC10100175; doi:10.1002/jnr.25148)
Supplement: Supplementary file 3 — Transparent Science Questionnaire for Authors [file JNR-101-316-s003.docx]

Rigorous study design and transparent reporting of results are the cornerstones of science. By maximizing the information provided in a manuscript, factors that may contribute to irreproducibility will be mitigated. The *Journal of Neuroscience Research* promotes transparency in research by strongly encouraging authors to include all relevant information about their studies (see our [preprint](https://osf.io/5cvqh/) for details). To expedite reviewer monitoring of these factors, authors submitting original research articles must complete this questionnaire.

If the manuscript is accepted and all items within the checklist are present, we will include a declaration of transparency at the end of the manuscript. This declaration reads as follows:

***The authors, reviewers and editors affirm that in accordance to the policies set by the Journal of Neuroscience Research, this manuscript presents an accurate and transparent account of the study being reported and that all critical details describing the methods and results are present***.

To complete the checklist, fill in the right-hand column with the page and paragraph number (e.g., ‘Page 3, Paragraph 2’) corresponding to the checklist item. If a checklist item is not applicable to the study being reported or the authors are unable to provide that item, a reason must be supplied. Additional comments can be added at the end of the document. Upload the completed document as supplementary information for review.

| Experimental and Study Design |  |
| --- | --- |
| 1. Clearly state the primary and any secondary objective or hypothesis of the study |  |
| 1. For each experiment, the study design must include:    1. Number of experimental and control groups    2. Randomization and blinding procedures and/or steps to minimize subjective bias when allocating subjects to experimental groups    3. Precise details of all procedures, including housing and husbandry are carried out in the experiment    4. Is sex considered as a biological variable? See [Editorial](https://pericles.pericles-prod.literatumonline.com/doi/full/10.1002/jnr.23979) for details about proper reporting | Page 6, Paragraph 2 |
|  | Using a tracking program |
|  | Methods; pages 6-9 |
|  | Yes, we studied both sexes |
|  |  |
| **Experimental Subjects** |  |
| 1. Specify the total number of subjects in each experiment, including the number of animals, sex and age in each group    1. Explain how the number of animals were arrived at and provide details of any sample size calculation, including power analysis    2. Indicate the number of independent replications of each experiment, when applicable | Page 6, Paragraph 2  Table 1 |
|  | Here, we studied the offspring of pregnant mice |
|  |  |
| **Data Handling** |  |
| 1. Indicate data collection start and stop rules:    1. Define the criteria for data/subject inclusion and exclusion. If any outcome or condition measure used was not reported in the results section, authors must address this omission    2. Specify reasons for any discrepancy between the number of animals at the beginning and end of the study    3. Define and explain how outliers are handled and report if data are removed prior to analysis |  |
|  | Not relevant |
|  | Illness or death of mice |
|  | 2 standard deviation |
|  |  |
| **Statistical Analysis and Depiction of Continuous Data** |  |
| 1. Provide details of the statistical methods used for each analysis    1. State, define and justify the statistical analysis used and specify the unit of analysis for each dataset    2. Describe and report methods used to assess whether data met the assumptions of the statistical approach and any adjustments for multiple comparisons    3. Fully report statistics (including exact value of N, degrees of freedom, test value and exact P-value when >0.001) and we encourage the use of effect sizes and confidence intervals    4. Disaggregated data are presented for males and females    5. Data distribution is depicted with univariate scatterplots boxplots, violin plots, or kernel density plots when presenting **continuous data** (see Editorial [Publishing Transparent and Rigorous Scientific Research](https://osf.io/5cvqh/)) |  |
|  | Page 9 Paragraph 2 |
|  |  |
|  | Results section and supplementary table 3 |
|  | Results section |
|  |  |
|  |  |
| **Discussion** |  |
| 1. Comment on study limitations including any potential source of bias, limitations to the animal model, imprecisions associated with the results, and the inability for any reason to study possible sex influences where they may exist. 2. Comment on possible translational implications and future research directions | Page 14 Paragraph 2 |
|  | Page 14 Paragraph 3 |
